# Gold-Assisted Molecular Imaging of Organic Tissue by MeV Secondary Ion Mass Spectrometry

**DOI:** 10.1021/jasms.3c00237

**Published:** 2023-09-08

**Authors:** Boštjan Jenčič, Paula Pongrac, Mirjana Vasić, Pia Starič, Mitja Kelemen, Marjana Regvar

**Affiliations:** † Jožef Stefan Institute, Jamova 39, 1000 Ljubljana, Slovenia; ‡ Biotechnical Faculty, University of Ljubljana, Jamnikarjeva 101, 1000 Ljubljana, Slovenia; § Jožef Stefan Institute Postgraduate School, Jamova 39, 1000 Ljubljana, Slovenia

## Abstract

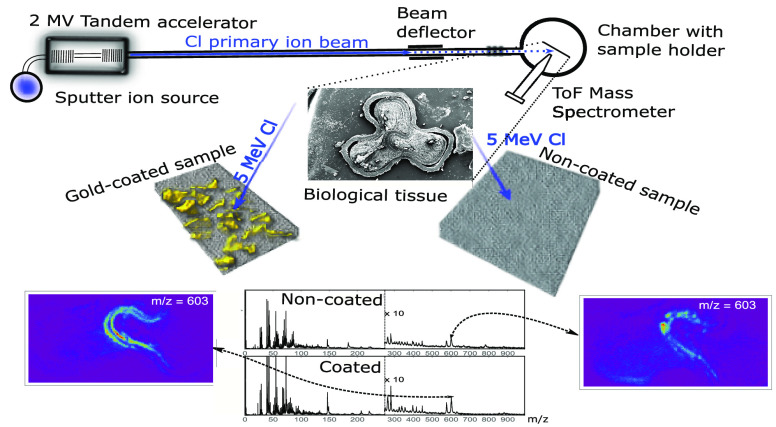

The quality of molecular imaging by means of MeV primary ion-induced secondary ion mass spectrometry by coating with gold was evaluated on different reference organic molecules and plant samples. The enhancement of the secondary ion yield was evident for the majority of the studied analytes, reaching the highest values at gold thicknesses between 0.5 and 2 nm, and increased the intensity up to 5-fold for reference samples and >2-fold for specific peaks within the plant sample. Improved propagation of the electric field due to the target potential on otherwise electrically insulating plant samples was also evident through improved image resolution and by reducing the background in mass spectra. However, detection of several molecules was significantly decreased at even at 1 nm thick gold layer. The results indicated that an optimized sequence of analysis is required to reliably interpret results.

## Introduction

Time-of-flight secondary ion mass spectrometry (ToF SIMS)^1^ is acknowledged for its prolific imaging capability, which is far exceeding most other mass spectrometry imaging techniques. Desorption of ionized secondary molecules through the interaction between the primary ion and the target results in significant undesired fragmentation of larger molecules and therefore reduces secondary ion yields for heavier (*m*/*z* > 1000) particles. Due to this characteristic, SIMS is a method of choice for analysis of inorganic materials, but its use is more demanding in organic samples.

An increase in the SIMS secondary ion yield can be achieved by (i) decreasing fragmentation, (ii) increasing sputtered material volume, or (iii) increasing ionization probability. Several approaches to achieve increased secondary ion yield have been proposed with different degrees of success.^2^ For example, the use of larger cluster ion beams, e.g., Bi_3_^+^, C_60_, or (H_2_O)_n_ clusters (*n* > 10000), has increased secondary ion yields for heavier (*m*/*z* > 200) molecules by several orders of magnitude, but resulted in spatial resolution penalty.^3^ Mixing an organic or nonorganic matrix into the sample to increase ionization probability, similar to the approach in matrix-assisted laser desorption ionization (MALDI), has also been proposed.^4^ Another approach is the so-called metal-assisted SIMS (MetA-SIMS), where coating samples with metals, such as gold and/or silver, has been demonstrated to improve the signal of observed heavier specimens.^5^

MetA-SIMS exhibited promising results with polymers,^5^ lipids,^6,7^ and other organic molecules.^8^ However, several studies reported the increase in secondary ion yield only with monatomic primary ions, while metal layers were counterproductive when employing polyatomic ions in most cases.^9−12^ Therefore, combining the secondary ion yield enhancement of heavy ion clusters and a metal coating is not always feasible. Still, in some specific cases, MetA-SIMS was demonstrated to be a useful tool even when using SF_5_^13^ or C_60_ primary ions.^14^

The mechanism behind MetA-SIMS is still not fully understood. The interaction between gold deposition on the sample and sample band structure may explain better sensitivity for fragments through freely accessible electrons.^5^ Other theories address the mitigation of matrix effects,^15^ analyte migration,^13^ and influence on metal layers on stopping power.^16^ The latter theory clarifies the difference in MetA-SIMS success with monatomic and polyatomic ions. While for the monatomic ions their impact is considered too violent, and some buffering is desired, polyatomic ions produce only shallow craters and cause suboptimal subsurface damage, whereby they do not reach the deeper lying analyte when covered by an additional metal layer.

MeV-SIMS is a variant of SIMS analysis,^17−19^ where the desorption mechanism, caused by MeV primary ions, relies on electronic excitations instead of nuclear collisions common for the keV energy range domain. In addition, MeV-SIMS induces less fragmentation of heavier organic molecules compared to conventional SIMS. Recent reports indicate MeV-SIMS is a promising stand-alone method, especially for organic matrices.^19,20^ The MetA-SIMS approach to increase secondary ion yield has not yet been studied in MeV-SIMS; therefore, the aim of the study was to test the effect of different thickness of gold layer deposited over organic material on the secondary ion yield in MeV-SIMS. Experiments were designed to test whether deposited gold layers will (i) affect secondary ion yields or (ii) present a beneficial conductive layer over an imperfectly smooth surface of an organic sample.

## Methods

### Sample Preparation

Four reference organic samples were examined: amino-acids glycine (*m*/*z* = 75), leucine (*m*/*z* = 131), arginine (*m*/*z* = 174), and hormone epinephrine (*m*/*z* = 183). All substances were purchased from Sigma-Aldrich and prepared as adequate solutions, which were deposited and spin-coated on roughly 1 × 1 cm^2^ silicon wafers. Arginine and glycine were dissolved in water with a concentration of 5 g 100 mL^–1^, while concentrations of leucine and epinephrine were 1 g 100 mL^–1^ Samples were coated with gold (coating machine BAL-TECH SCD 005) of various equivalent thicknesses (volume per area unit), namely 0.5, 1, 2, 3, 4, 5, 6, 7, and 9 nm. Coating with the aforementioned thicknesses of gold, especially those under 5 nm, could not be done homogeneously; therefore, a parameter of thickness equivalent was used. Plasma coating proceeded in a vacuum of approximately 5 × 10^–2^ mbar for duration between 5 and 90 s, working distance of 50 mm and sputtering current 20 mA. Prior to each coating, samples were flushed with argon.

Analyzed biological tissue was a cross section of Tartary buckwheat (*Fagopyrum tataricum*) grain. The samples were prepared by soaking the grain at 5 °C for 2 h, hand-sectioned to approximately 1 mm thick sections using a sharp platinum-coated razor blade, placed between several layers of tin foil, frozen in liquid nitrogen and freeze-dried as described in detail previously.^21^

After the MeV-SIMS analysis was completed (see below), grain cross sections were coated with gold of 1 nm equivalent thickness and analyzed again with MeV-SIMS. Before imaging the cross sections with scanning electron microscopy (SEM, Thermo Fisher Quanta 650 ESEM), samples were coated with 5 nm of gold.

### MeV-SIMS Analysis

MeV-SIMS was performed at the Microanalytical center of Joef Stefan Institute, Ljubljana, Slovenia. The 2 MV tandem accelerator was used to accelerate ^35^Cl^5+^ primary ion beam with the energy of 5.0 MeV. The intensity of the beam on the target varied between 60 and 300 pA, depending on the settings of the collimator and object slit apertures, which were used for rough focusing and shaping of the primary ion beam. In order to conduct ToF measurement of desorbed secondary ions, a primary ion beam was pulsed with a frequency of 10 kHz. On average, each pulse generated between 0.2 and 1.0 primary ions, resulting in a primary ion intensity between 2 and 10 kHz (range of fA). Chlorine beam was focused to its final dimensions of approximately 10 × 10 μm^2^ by means of magnetic quadrupole triplet lenses.

Each ToF measurement cycle lasted 100 μs, and secondary ions were analyzed on the linear side of a dual type (linear + reflectron) ToF spectrometer. The length of the linear side was 1.0 m, and the accelerating voltage was 3 kV. Samples were analyzed in a vacuum chamber with a back pressure of 5.0 × 10^–8^–1.5 × 10^–8^ mbar. Mass resolving power m/dm was estimated to be approximately 500 for the peak of the protonated arginine molecule (*m*/*z* = 175).

Analysis of each reference sample was performed on three different spots more than 1 mm apart from each other. The analyzed area was 300 × 200 μm^2^, and a duration of each measurement was 5 min. Samples were bombarded by 2000–4000 primary ions per second, which corresponds to a primary ion dose in the range of 10^9^ ions/cm^2^, similar to that used for biological tissue.

Grain cross sections were first analyzed noncoated. Analysis lasted over the night (approximately 12 h). Primary ion count rate was monitored by the channel electron multiplier (CEM) detector before the start and after completion of analysis. At both times, it was approximately 2500 primary ions/s ± 100 ions/s; hence, stable measuring conditions were assumed. The scan size was 2000 × 1200 μm^2^; therefore, the total primary ion fluence on the sample was approximately 5 × 10^9^ ions/cm^2^, almost three magnitudes of order less than the static SIMS limit, at which chemical alteration of the sample is expected. Such a low primary ion dose allowed good multiple measurements of the same sample with 256 × 256 pixels image resolution. Afterward, the samples were coated with a gold thickness equivalent of 1 nm, a value that exhibited greatest effects with reference samples. The same position of the sample was analyzed again for the same duration with a similar primary ion count rate of 2200 ± 100 ions/s to achieve the highest possible similarities between the two measurements.

## Results and Discussion

### Secondary Ion Yield Enhancement in Reference Organic Samples

**Figure 1:**
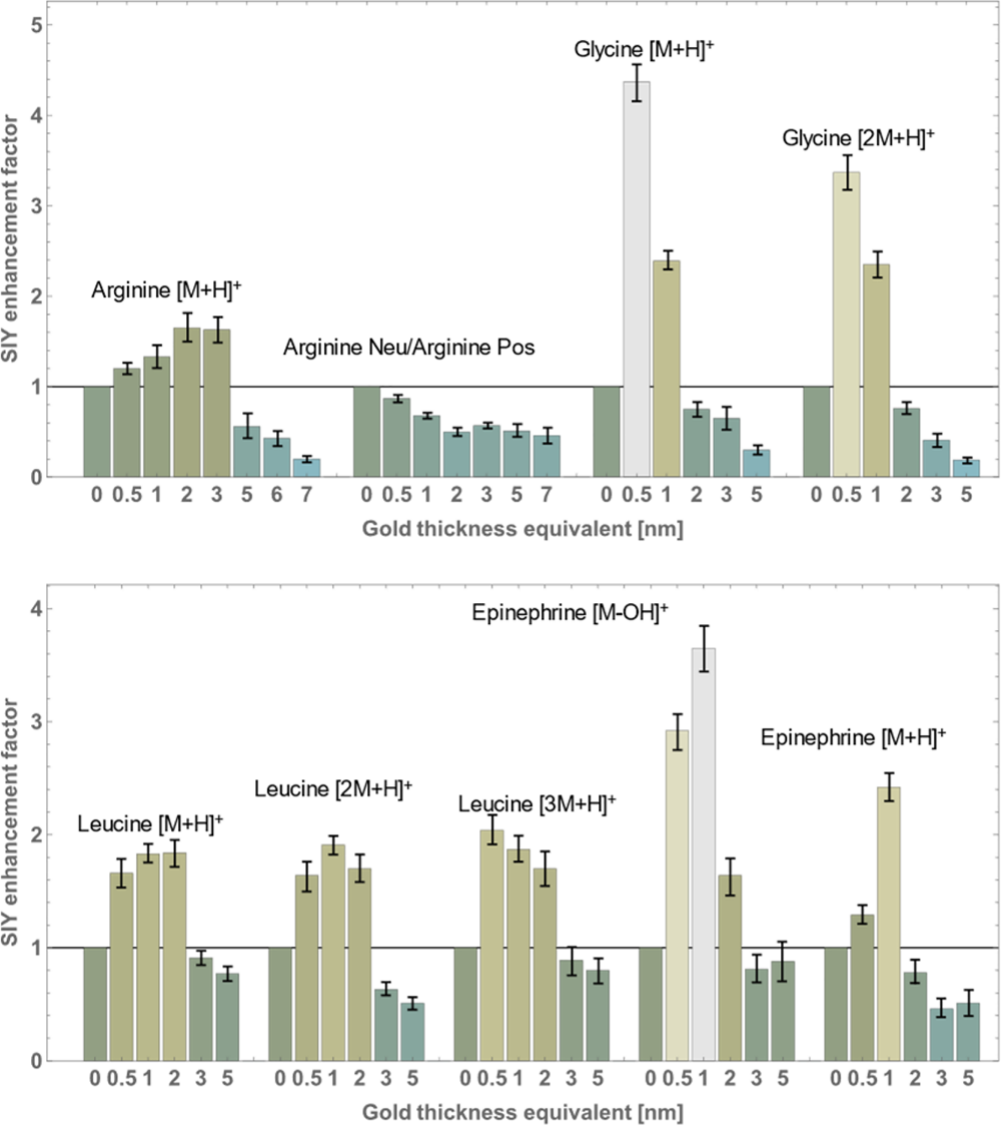
Secondary ion yield (SIY) enhancement factor as a function of gold thickness equivalent of different molecular peaks [*n*M + H]^+^ and [M – OH]^+^. Thickness equivalents of 0.5 and 1.0 nm resulted in SIY increase for all observed samples, 2 and 3 nm yielded different results, while above 5 nm gold thickness equivalent all intensities were reduced.

Secondary ion yields for different reference organic samples depended on the gold thickness in a similar, yet not identical, manner. In general, gold-coating increased the secondary ion yield for all measured reference samples (Figure 1). The maximum average enhancement factor (defined as secondary ion yield of coated sample divided by secondary ion yield of noncoated sample) of three measurements for selected samples varied between 1.6 and 4.4. Variations of secondary ion yield between measurements of the same targets with the same gold thickness were in the range between 10 and 20%. Secondary ion yield reached maximum at different thickness equivalents between 0.5 and 2 nm and generally decreased at and above 5 nm.

For arginine, the highest increase for the secondary ion yield was observed at 2 and 3 nm gold thickness equivalents, and the measured enhancement was 1.6-fold. Secondary ion yield increased from 4.2 × 10^–2^ to 6.9 × 10^–2^ secondary ions/primary ion. Typically, all measurements of noncoated reference samples resulted in secondary ion yields ranging between 10^–2^ and 10^–1^ secondary ion/primary ion.

All observed leucine peaks (M + H, 2M + H, and 3M + H) exhibited similar dependence on the gold thickness as those for arginine. Their maxima were between 0.5 and 2.0 nm gold thickness equivalent, and the enhancement factors were 1.8 (M + H), 1.9 (2M + H), and 2.0 (3M + H). Each subsequent cluster has approximately four times less intensity, with 7M + H being the heaviest detectable cluster when analyzing the sample for a duration of 300 s.

For glycine, on the other hand, both M + H and 2M + H peaks had the highest increase with 0.5 nm gold thickness equivalent. M + H secondary ion yield increased by a factor of 4.4 and 2M + H by a factor of 3.4. It was evident in glycine spectra that M+H peak was increased by more than cluster peaks and later decreased less with thicker gold layers.

Two intense peaks in the epinephrine spectra, M – OH and M + H, reached their maxima at 1 nm gold thickness equivalent to the 3.7- and 2.4-fold increase, respectively.

By coating the reference organic samples with gold, only the intensity of already prominent protonated (quasi)molecular peaks increased. In addition, there was no apparent alteration of the spectra, apart from the occurrence of few peaks at low *m*/*z* regions, attributable to contamination. Cationization in the form of M + Au was not observed in the positive mode, nor were additional peaks observed in the negative mode. Therefore, further detailed analysis was only conducted on positive ions, which are being analyzed much more frequently. There were also no gold clusters observed, which are commonly detected in MetA-(keV) SIMS spectra.^5^ Thus, molecular fingerprints in gold-coated samples did not differ from those in noncoated samples, but had better peak-to-background ratio, making the identification of peaks easier and interpretation of results simpler.

For the arginine peak, the amount of metastable decay as a function of gold thickness was also measured to gain valuable information on the internal energy of sputtered ionized molecules (also shown in Figure 1). To this end, additional positive voltage was applied on the electrode in front of the MCP detector. This voltage was higher than the acceleration voltage; therefore, only neutral particles (fragments of metastable decay) were detected by the MCP detector.

Our previous findings^22^ have displayed the importance of post desorption decayed fragments on the quality of mass spectra, since these product ions have slightly different energy than the original (precursor) ions, and can be observed as a “background” by the molecular peak. It has been shown that, by increasing primary ion energy and, consequentially, increasing the electronic stopping power contribution, the amount of such fragments decreases. New results with coated samples show lower amounts of decayed neutrals (in comparison to normal positive signals) with an increase in gold-layer thickness. Coating with gold appears to reduce the intensity of product ions (fragments) linearly up to a thickness equivalent to 2 nm of gold layer, where neutral signal was decreased to approximately 50% in relation to positive signal. With thicker gold layers, this ratio remained stable.

### Analysis of Organic Tissue

**Figure 2:**
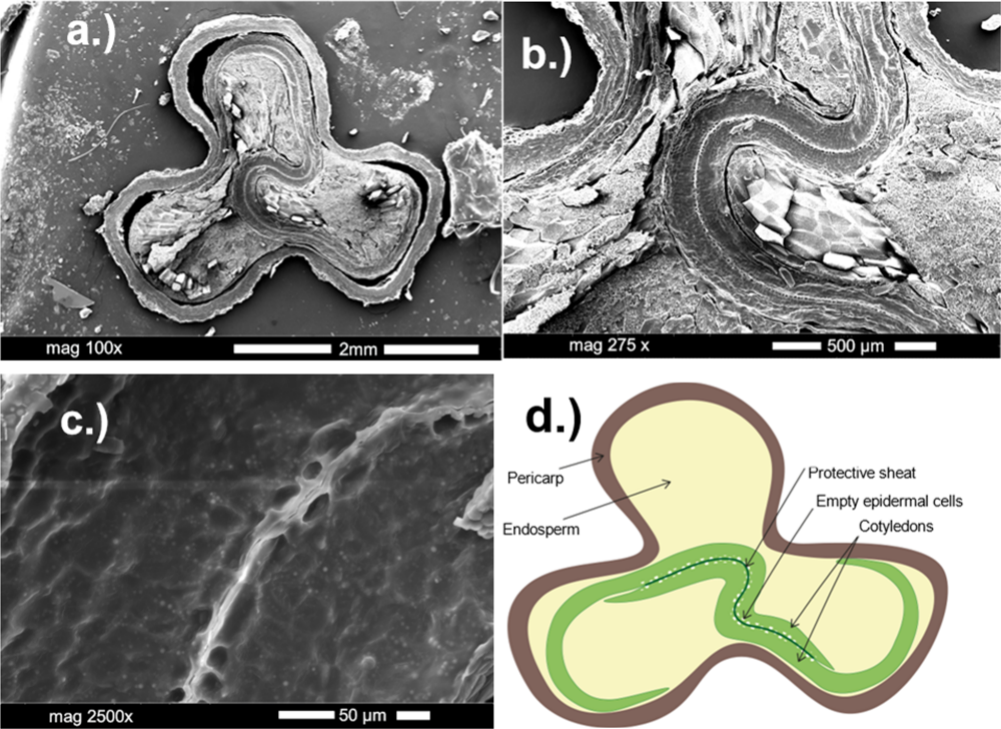
Scanning electron micrographs of Tartary buckwheat grain cross section. (a) Whole sample, (b) central part of the grain where two fitting cotyledons wind among endosperm cells, (c) close-up of the cotyledons with empty epidermal cells in the middle, and (d) scheme of the sample. Sample was coated with an additional 5 nm thick layer of gold for SEM images.

**Figure 3:**
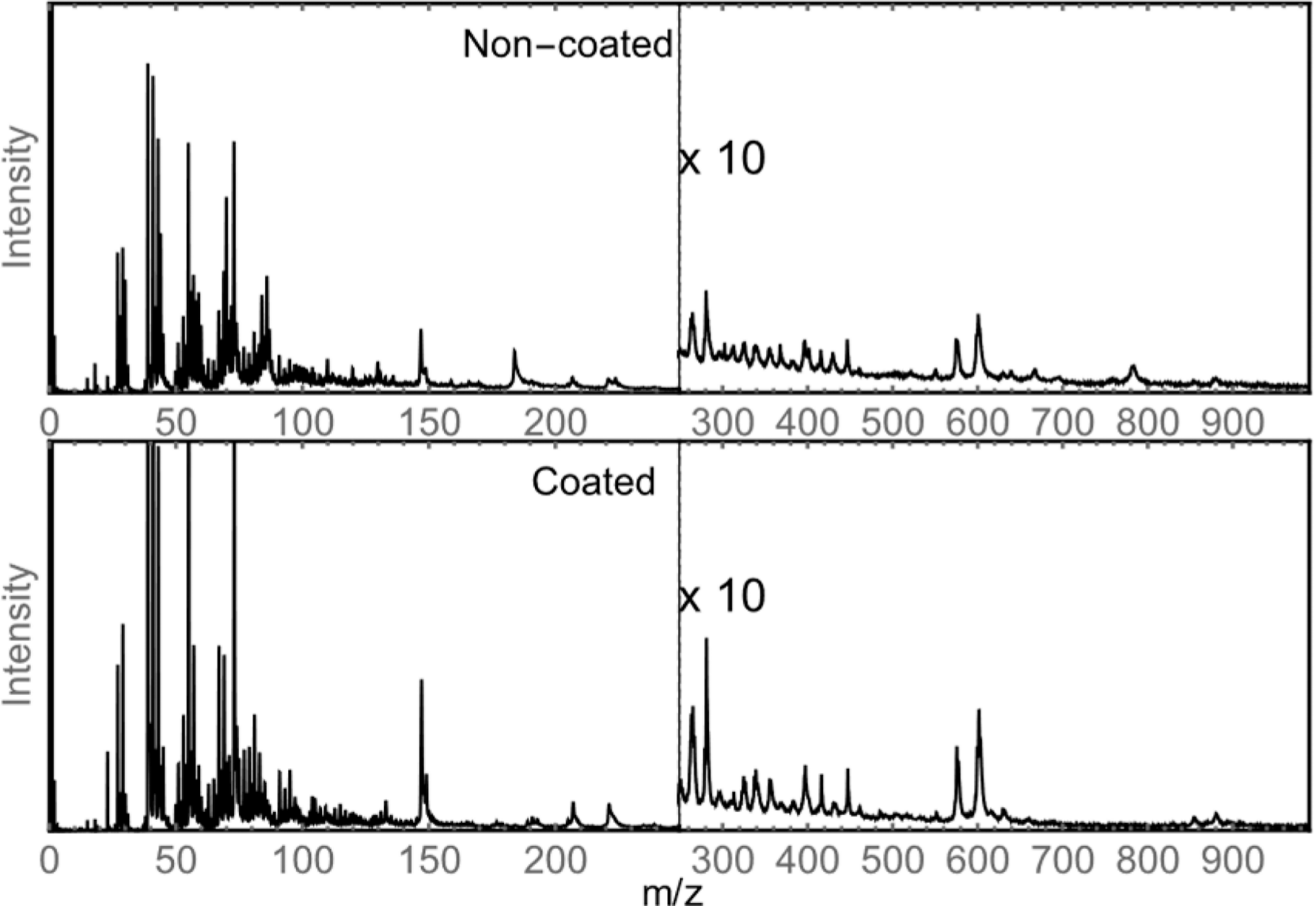
MeV-SIMS spectra of noncoated and 1 nm gold coated Tartary buckwheat grain. Spectra were normalized to the primary ion dose. Coating of the sample with gold resulted in apparent contamination of the spectra in the low mass region, with the intensity of several peaks (e.g., *m*/*z* = 23, 39, 41, 43, 55, 57, 70, 73, and 147 and between *m*/*z* = 300 and 1000) being much higher compared to that for noncoated grain.

The selected organic tissue was Tartary buckwheat grain, which in a cross section resembles three-leaved clover and has three major tissues: outer husk, inner starch-rich endosperm, and two winding cotyledons (Figure 2). To test the effect of gold coating on the secondary ion yield, the cross section was analyzed directly, after which it was (based on above-discussed results) coated with 1 nm gold thickness equivalent and analyzed again with only a minor offset. The cumulative spectra of noncoated and gold-coated grain confirmed results from reference organic samples; namely, the peak height was improved when the grain was coated with gold (Figure 3).

The spectrum of the gold-coated sample displayed a considerably higher signal of some low-mass peaks, such as *m*/*z* = 23, 39, 41, 43, 55, 70, 73, and 147. The onset of these peaks can be at least partly attributed to contamination during coating or during transport since these peaks prominently emerged in spectra of reference samples as well. Maps of these peaks are consequently not tissue specific, even if they were before coating. Here, the main issue comes with peak at *m*/*z* = 39, namely, potassium (K^+^), whose concentration in plant samples is typically among the highest and whose allocation can be used to distinguish organic tissue from the surrounding material. With adding gold, *m*/*z* = 39 maps lose such function. Either the additional signal could be more K contamination, or it could be some organic fragment such as C_3_H_3_^+^, which cannot be distinguished with available mass resolution. For the purpose of tissue recognition, we afterward used hydrogen or total ion count maps.

**Figure 4:**
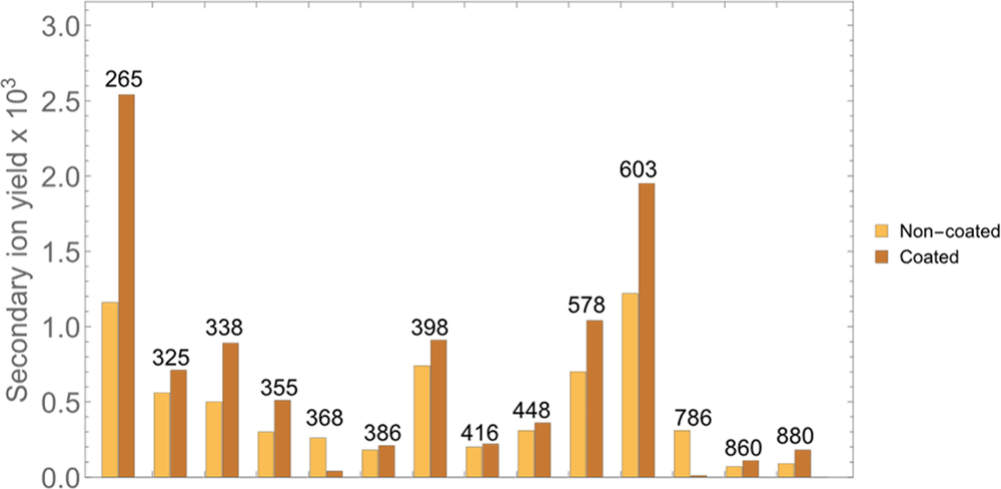
Secondary ion yields for selected peaks within the MeV-SIMS spectrum of Tartary buckwheat grain either coated with 1 nm gold or not. The increase in intensity ranges between 1.2- and 2.2-fold, except for two peaks (*m*/*z* = 368 and *m*/*z* = 786), where a significant suppression of the signal was observed in the coated sample.

In the higher mass domain, the majority of peaks could be attributed to a specific tissue. Figure 3 depicts the increase in their intensity and also the decrease in the background. Most of the tissue specific peaks were increased by a factor between 1.2 to 2.2, similarly to enhancement observed for the reference organic samples. Comparisons of secondary ion yields for tissue-specific peaks are presented in Figure 4. Secondary ion yields were mostly in the range of 10^–4^–10^–3^, an expected rate given the lower concentration in relation to reference materials. Also, these values correspond to average secondary ion yields over the whole scan. Secondary ion yield for the peak at *m*/*z* = 603 was 1.22 × 10^–3^ secondary ions/primary ion and increased to 1.95 × 10^–3^ secondary ion/primary ion after gold coating. However, within the selected area with 6232 pixels (9.5% of scanned area), the secondary ion yield of this peak was 0.76 × 10^–2^, and it increases to 1.33 × 10^–2^ secondary ions/primary ion after coating.

For specific peaks, the signal from the coated ample was almost completely suppressed. Such was the case for the peak at *m*/*z* = 786, with intensity falling to only 3% of the one in the noncoated case, and the peak at *m*/*z* = 368, where intensity is decreased to 16% of the previous value.

**Figure 5:**
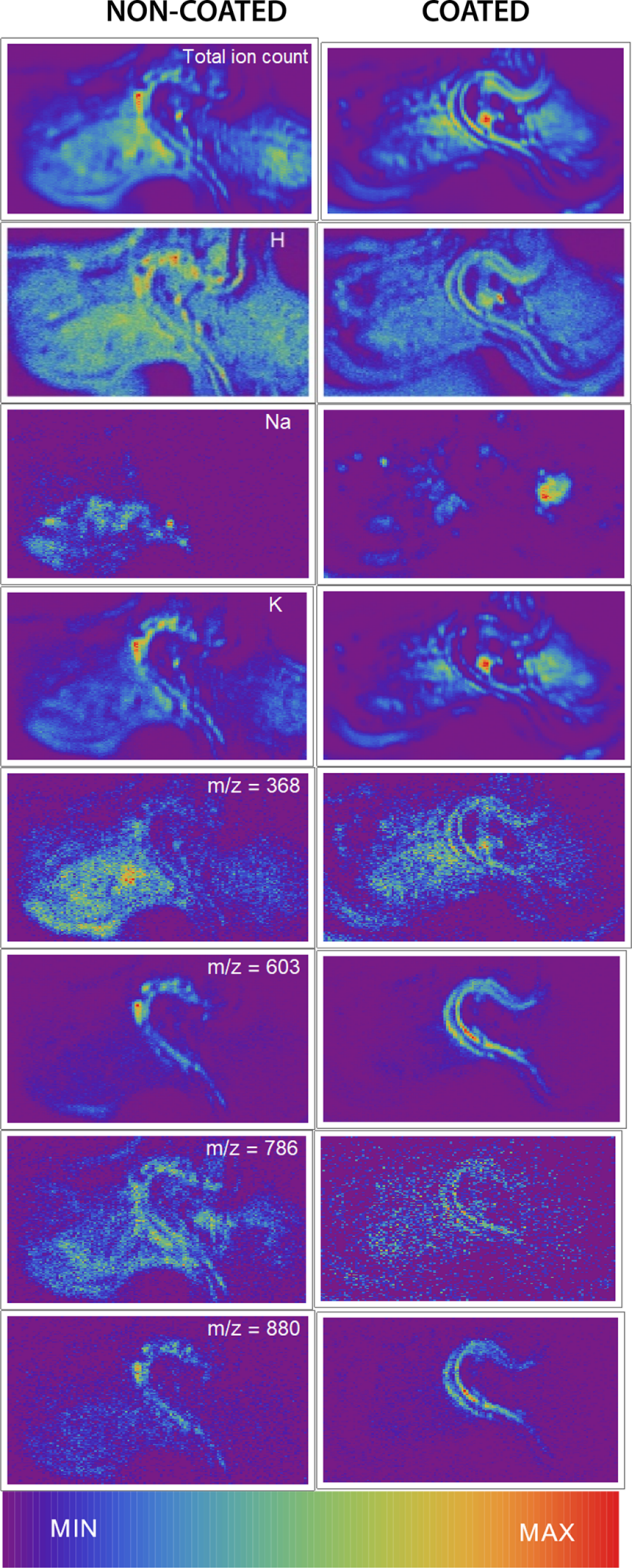
Selected molecular maps of Tartary buckwheat grain for noncoated (left) and gold-coated (right) sample. Na and K maps are two examples within the low mass region, where there is no correlation between maps of noncoated and coated samples. Hydrogen maps, on the other hand, can still be used to recognize tissue morphology even after coating. Peaks above *m*/*z* = 200 exhibit three prevalent distributions in the noncoated sample: Cotyledon localized (*m*/*z* = 603 and 880), endosperm localized (*m*/*z* = 368), and widespread (*m*/*z* = 786). The three distributions translate differently after coating, with the first one keeping the highest similarity to noncoated maps. Scan size: 2000 × 1200 μm^2^.

The effect of the gold-coating on the molecular imaging of the Tartary buckwheat grain can be visualized in Figure 5. Scan size: 2000 × 1200 μm^2^. Images were stretched along the *x*-axis due to the rotation of the sample in relation to the primary ion beam axis.

Hydrogen or K (*m*/*z* = 39) maps of the noncoated sample reveal basic parts of the Tartary buckwheat grain: cotyledon, endosperm, and pericarp. Sodium, on the other hand, appears to be present in the form of crystals within the endosperm and has a unique distribution. Among peaks with *m*/*z* > 200, three general types of distributions were distinguished. The first distribution appeared most tissue specific and was observed for peaks at *m*/*z* = 603 and *m*/*z* = 880; the majority of the signal in this distribution type arises from the cotyledons. In addition, peaks whose distribution is similar have *m*/*z* values of 265, 325, 338, 355, 386, 398, 413, 448, 578, and 860.

The second distribution type was observed for molecules under peaks *m*/*z* = 368, for which a significant amount of signal comes from the 150 × 150 μm^2^ spot in the endosperm. Similar distribution was also observed for peaks at *m*/*z* = 403, 430, 640, and 666. Lastly, the third distribution type was observed for the peak at *m*/*z* = 786, which seemed to have a somewhat unique distribution, which is still concentrated to the cotyledon, yet much of the signal also comes from elsewhere.

After coating, maps of the first distribution type remained very similar to that found in noncoated samples, with majority of the signal being allocated to the cotyledon. However, maps of the coated sample resemble the expected distribution within the cotyledon better with homogeneous signal on sides and no signal coming empty epidermal cells, visible in Figure 2. Among these tissue specific peaks, at least modest increase of their intensity was observed. Maps of noncoated and coated samples therefore differ in two aspects: (i) the background was significantly higher with the noncoated sample, and (ii) the sample topography seems to have much less of an impact when the sample was gold-coated. For both differences, we can assume that the target voltage application was inadequate for the noncoated sample due to its thickness and due to the insulating nature of the plant tissue.

The peaks from the second distribution type had a different response to gold coating, with some decreasing in intensity and some remaining stable. Their maps did not keep the same characteristics as was the case with the first group. Similarly, the peak at *m*/*z* = 786 lost most of its intensity after coating, and its map was afterward correlated to the background only.

**Figure 6:**
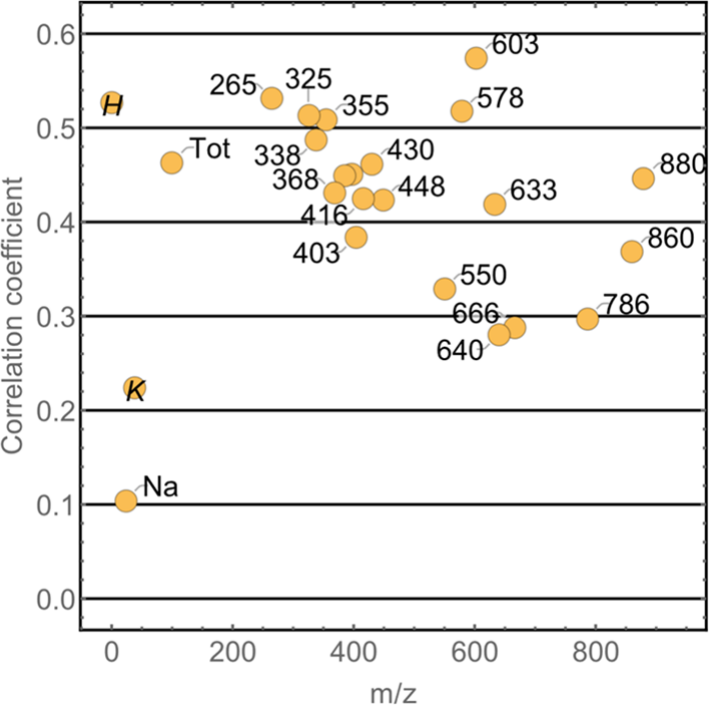
Correlation coefficients of selected peaks for maps of coated and noncoated Tartary buckwheat grain. Na and K maps exhibit no correlation (below 0.25), while tissue specific peaks have a moderate correlation (around 0.5). Correlation was reduced mostly because of the varying impact of sample topography and background.

Figure 6 displays correlation coefficients for coated and noncoated maps. In order to obtain the best possible match of coated and noncoated maps, we have rotated and translated maps of the coated sample. Through the method of least-squares, we have calculated the optimum rotation angle of 0.04 rad (approximately 2.3°) and translation vector (0,4) pixels. The calculated stretches in the *x* and *y* directions were between 0.99 and 1.01 and were neglected. This was expected since the two measurements had the same scan size and angle alterations were minimal.

As can be seen in Figure 6, most peaks from the group with the sample specific distribution had the highest correlation coefficient, typically approximately 0.5, which is commonly recognized as moderate correlation. It is obvious from Figure 5 that the two types of maps are not highly correlated even for these peaks, since the signal in the noncoated sample is more dependent on the sample’s topography. The correlation coefficient was also positively correlated with background. Some more prominent peaks, such as *m*/*z* = 603 or 578, have better correlation simply because of the lesser impact of the background. On the contrary, calculations for peaks at *m*/*z* = 860 or 880 yielded significantly lower correlations, although the translation of their maps from the noncoated to coated case seems very similar to the one in the *m*/*z* = 603 case.

It is not surprising that the correlation coefficients of the second and the third distribution groups are significantly smaller, mostly approximately 0.3, which is considered a very limited correlation. Even lower correlations can be observed for K and Na maps, which exhibit no similarities. On the other hand, hydrogen and total ion count maps are also moderately correlated.

## Conclusion

The enhancement of molecular imaging using MeV-SIMS after the gold coating of organic materials was demonstrated. Analysis of all reference organic samples has emphasized the positive effect of coated metal on secondary ion yield, similar to the case with keV-SIMS. However, with MeV primary ions, the change in primary ion energy after penetrating gold layers is minimal (energy loss of approximately 5.6 keV/nm), so the effect of changed stopping power cannot be considered. Other favored factors, such as analyte migration and mitigation of matrix effects, might be the main mechanisms behind the secondary ion yield increase. Despite the secondary ion yield, enhancement did not exceed a factor of 5, which is much lower than that with some other sample alteration techniques, such as salt treatment.^23^ This result is still promising, since preparation protocols are very straightforward. The main benefit of “MetA-MeV-SIMS” is still the advancement of imaging capabilities, especially when thicker insulating samples are used. Besides improving secondary ion yields, coated samples were measured with an additionally better peak-to-background ratio, and the effects of sample topography have been reduced.

By contrast, interpretation of results was not straightforward, Novel peaks in the low mass region were prominent and generally prevented reliable molecular imaging below *m*/*z* = 100. There were also some additional contaminants in the mass range of 100–300 u, mostly from poly(dimethyl siloxan) (PDMS) oligomers. In order to optimize the sample coating protocol, better vacuum (in the range of 10^–5^ mbar or lower) is needed during coating. Additional discrepancies between the spectra of noncoated and coated samples were also observed, such as suppression of several peaks. However, these peaks might not be sample specific and could only be surface contaminants, which were removed from the sample after argon rinsing.

Considering all the advantages and uncertainties of gold coating for MeV-SIMS measurements, analysis of coated samples can be best utilized in combination with analysis of pure samples. With MeV-SIMS, small primary ion fluences of approximately 10^10^ ions/cm^2^ have been demonstrated to map biological tissue with sufficient statistics. Repeating the measurement with coated samples is feasible due to low induced damage of such ion beam fluence and gives additional information; therefore, such sample treatment may be best utilized as an addition to the conventional analysis.
